# KDAC8 with High Basal Velocity Is Not Activated by N-Acetylthioureas

**DOI:** 10.1371/journal.pone.0146900

**Published:** 2016-01-08

**Authors:** Tasha B. Toro, Subramanya Pingali, Thao P. Nguyen, Destane S. Garrett, Kyra A. Dodson, Kyara A. Nichols, Rashad A. Haynes, Florastina Payton-Stewart, Terry J. Watt

**Affiliations:** Department of Chemistry, Xavier University of Louisiana, 1 Drexel Dr., New Orleans, Louisiana, United States of America; Saint Louis University School of Medicine, UNITED STATES

## Abstract

Lysine deacetylases (KDACs) are enzymes that reverse the post-translational modification of lysine acetylation. Recently, a series of N-acetylthioureas were synthesized and reported to enhance the activity of KDAC8 with a fluorogenic substrate. To determine if the activation was general, we synthesized three of the most potent N-acetylthioureas and measured their effect with peptide substrates and the fluorogenic substrate under multiple reaction conditions and utilizing two enzyme purification approaches. No activation was observed for any of the three N-acetylthioureas under any assayed conditions. Further characterization of KDAC8 kinetics with the fluorogenic substrate yielded a k_cat_/K_M_ of 164 ± 17 in the absence of any N-acetylthioureas. This catalytic efficiency is comparable to or higher than that previously reported when KDAC8 was activated by the N-acetylthioureas, suggesting that the previously reported activation effect may be due to use of an enzyme preparation that contains a large fraction of inactive enzyme. Further characterization with a less active preparation and additional substrates leads us to conclude that N-acetylthioureas are not true activators of KDAC8 and only increase activity if the enzyme preparation is below the maximal basal activity.

## Introduction

Lysine deacetylases (KDACs, also known as histone deacetylases, EC 3.5.1.98) are enzymes that reverse the post-translational modification of lysine acetylation, by catalyzing the hydrolysis of ε-N-acetyllysine residues in proteins via a conserved mechanism.[1–3] This cycle of acetylation and deacetylation has been linked to many biological processes, including development and growth, memory formation, and regulation of metabolism.[4–7] KDAC activity has also been linked to numerous diseases, in particular chronic diseases such as asthma, cancers, muscular disorders, and diabetes.[7–10] KDACs are commonly grouped into several classes, with class I, II, and IV KDACs being metal-dependent, and class III (sirtuins) being NAD-dependent. Over 1000 inhibitors for KDACs have been identified, and several are in clinical trials or have already been approved for therapeutic use.[9,11]

There has also been considerable interest in identifying small molecules that activate KDACs, increasing activity in the presence of a substrate, in particular because most KDACs are relatively slow enzymes under *in vivo* conditions.[12] A variety of natural products have been identified that stimulate activity of one or more KDACs.[13–16] Recently, several N-acetylthioureas were identified as synthetic activators of KDAC8 with high selectivity and potency, with a rate enhancement of up to 20-fold.[17,18]

Here, we evaluate the ability of three N-acetylthiourea molecules to activate KDAC8 under multiple reaction conditions and with multiple substrates. Our findings suggest that these molecules do not activate KDAC8 unless the enzyme preparation has inherently low activity.

## Experimental

### Synthesis of N-acetylthioureas

Synthesis was performed as previously described.[17] Briefly, 7.1 mmol of benzoyl chloride were added over 5 min to 7.8 mmol of room-temperature NH_4_SCN in 10 mL acetone and the mixture was heated at reflux for 30 min. Heating was stopped and 7.1 mmol aryl amine (aniline, 3,5-dimethoxyaniline, or methylbenzylamine for TM-2-51, TM-2-88, or TM-2-104, respectively) in acetone were rapidly added to maintain a vigorous reflux. The mixture was heated for an additional 30 min to maintain reflux. The product was isolated by pouring the mixture over cracked ice with vigorous stirring until the ice melted, and the resulting solid collected by filtration. The solid was washed with water, cold water/methanol (1:1), and methanol, then dried at room temperature. TM-2-104 was further purified by silica gel chromatography and extensively dried to yield the expected oil. Yields of the final products were 60–80%. Stock solutions were prepared at 10 mmol L^-1^ in DMSO and stored at -20°C.

### Expression and purification of KDAC8

For most assays, KDAC8 was expressed and purified as previously described.[19] Briefly, KDAC8 was expressed in BL21(DE3) cells using a T5 promoter, then purified by metal affinity chromatography with Talon cobalt resin (Clontech) using a batch/column method. Protein was washed in 30 mmol L^-1^ MOPS pH 8.0, 150 mmol L^-1^ KCl, and 5.0 mmol L^-1^ imidazole, then eluted in the same buffer containing 150 mmol L^-1^ imidazole. Following initial purification, the His_6_ tag was cleaved using tobacco etch virus protease during dialysis and the resulting enzyme isolated by eliminating contaminants using a second metal affinity column. Enzymes were stored in 30 mmol L^-1^ MOPS pH 8.0, 150 mmol L^-1^ KCl, 25% glycerol, and 1 mmol L^-1^ tris(2-carboxyethyl)phosphine. Where noted, KDAC8 was expressed identically then purified using Ni Superflow Resin (Clontech) instead of cobalt resin. This method was identical to that previously described except that wash buffer contained 20 mmol L^-1^ imidazole, elution buffer contained 300 mmol L^-1^ imidazole, and the tag cleavage step and second column were skipped; protein was dialyzed directly into the storage buffer following the initial purification. All assays described below were performed using multiple, independently expressed batches of enzyme. KDAC8 expressed in a baculovirus system with a C-terminal His_6_ tag was purchased (BPS Bioscience) and dialyzed into 30 mmol L^-1^ MOPS pH 8.0, 150 mmol L^-1^ KCl, and 25% glycerol, then stored in the same buffer with addition of 1 mmol L^-1^ tris(2-carboxyethyl)phosphine. Protein purity was assessed by SDS-PAGE and stained with GelCode Blue (Thermo Scientific).

### Fluorescamine assay

Peptides were synthesized (Genscript) and purified to > 95%. Reaction conditions were as previously described.[19] Briefly, 200 nmol L^-1^ KDAC8 and 100 μmol L^-1^ peptide were incubated in assay buffer 1 (30 mmol L^-1^ potassium phosphate pH 7.6, 100 mmol L^-1^ KCl, and 5% glycerol) for 1 hr at 25°C in the absence or presence of 10 μmol L^-1^ N-acetylthiourea, stopped with 100 μmol L^-1^ suberoylanilide hydroxamine (SAHA), and product formation was monitored by addition of fluorescamine. Errors represent the standard deviation of three independent experiments.

### Fluor-de-Lys assay

Reaction conditions were as previously described,[17] with the following variations. 200 nmol L^-1^ KDAC8 and 100 μmol L^-1^ Fluor-de-Lys substrate (Enzo Life Sciences) were incubated in assay buffer 1, assay buffer 2 (50 mmol L^-1^ tris pH 8.0, 137 mmol L^-1^ NaCl, 2.7 mmol L^-1^ KCl, and 1 mg mL^-1^ BSA), or assay buffer 3 (30 mmol L^-1^ MOPS pH 8.0, 150 mmol L^-1^ KCl, and 5% glycerol), as noted, for 1 hr at 25°C in the absence or presence of 10 μmol L^-1^ N-acetylthiourea. Reactions with the Fluor-de-Lys HDAC8 substrate (Enzo Life Sciences) utilized 150 nmol L^-1^ KDAC8 and 150 μmol L^-1^ substrate incubated in assay buffer 4 (25 mmol L^-1^ tris pH 8.2, 137 mmol L^-1^ NaCl, 2.7 mmol L^-1^ KCl, and 1.0 mmol L^-1^ MgCl_2_) for 10 min at 25°C in the absence or presence of 100 μmol L^-1^ TM-2-51. Reactions were stopped by addition of SAHA to a final concentration of 100 μmol L^-1^ and trypsin to a final concentration of 1 mg mL^-1^, then incubated at 37°C for 15 min before being read in a 96 well plate with 360 nm excitation and 460 nm emission at 25°C. Fluorescence was converted to activity using a standard curve of the free 7-amino-4-methylcoumarin. Where noted, Zn^2+^ or Co^2+^ (ICP-MS standard quality, Ultra Scientific) were pre-incubated with the enzyme prior to addition of substrate. Errors represent the standard deviation of three to six independent experiments, except where noted.

### Steady-state kinetics

140 nmol L^-1^ KDAC8 and various concentrations of Fluor-de-Lys substrate were incubated as described for the Fluor-de-Lys assay in assay buffer 2. Aliquots were mixed with SAHA to stop reactions at 0, 15, 30, and 45 min, then all aliquots were treated with trypsin simultaneously. The resulting data was non-linearly fit to the Michaelis-Menten equation as previously described.[19]

## Results

**Fig 1:**
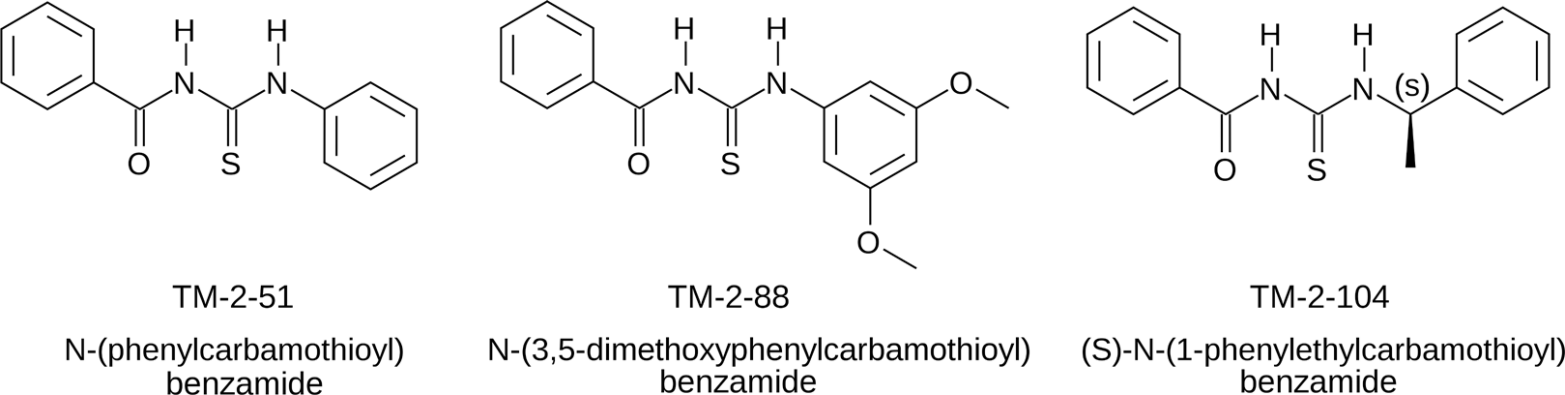
Structures of the N-acetylthioureas evaluated. Molecules were synthesized as described in the Experimental section, and data validating synthesis is provided as supplemental information.

The synthetic N-acetylthiourea products (Fig 1) match the data previously reported for the molecules except for the colors of the solids TM-2-51 and TM-2-88. These powders were originally described as yellow and white, respectively,[17] but we observed the colors to be swapped. However, all NMR and melting point data on our synthesized molecules agree with the previous report (S1–S4 Tables). To determine the effect of the N-acetylthioureas on the activity of KDAC8 with peptide substrates, deacetylation of peptides was evaluated in the presence or absence of the N-acetylthioureas. The peptides used here have been previously shown to be substrates for KDAC8.[19,20] As shown in Table 1, the N-acetylthioureas failed to increase activity when added to the reaction at 10 μmol L^-1^. Increasing the concentration of each N-acetylthiourea to 100 μmol L^-1^ also failed to increase enzyme activity above the activity level observed in the absence of the molecules.

**Table 1:** Effect of N-acetylthioureas on KDAC8 activity with peptide substrates in assay buffer 1.

	Activity (pmol min^-1^ μg^-1^)			
Substrate	No N-acetylthiourea	10 μmol L^-1^ TM-2-51	10 μmol L^-1^ TM-2-88	10 μmol L^-1^ TM-2-104
ac-FR-{K-ac}-RW-am	21.0 ± 1.3	20.3 ± 2.1	19.6 ± 1.9	19.7 ± 1.4
ac-IS-{K-ac}-FD-am	10.0 ± 2.6	9.9 ± 2.7	9.8 ± 2.9	9.9 ± 2.7

To evaluate if the effect of the N-acetylthioureas was specific to the Fluor-de-Lys substrate and/or particular buffer conditions, we performed the Fluor-de-Lys assay with and without N-acetylthioureas present, in both the phosphate buffer used with the peptide substrates and the standard Fluor-de-Lys buffer as previously reported. Again, we did not observe any significant activation of the enzymes by the N-acetylthioureas (Table 2). To ensure that the lack of effect was not due to the different purification protocol that we utilized,[19] we purified another batch of KDAC8 using Ni Superflow resin similarly to the method previously described.[17] The enzyme purified on nickel resin was significantly less pure than that purified by our standard method (S1 Fig). The resulting protein was also unaffected by N-acetylthioureas (Table 2), and exhibited approximately 4-fold lower activity than the enzyme prepared using our standard method. To determine if this lack of activation was independent of our enzyme purification process, we performed the same experiments with a commercial preparation of KDAC8. The baseline activity of this preparation was lower, but statistically significant 1.5- to 2-fold activation was observed (Table 2). Both spectroscopic methods and gel electrophoresis (S1 Fig) were used to confirm that the concentrations of enzyme used in these reactions were the same.

**Table 2:** Effect of N-acetylthioureas on KDAC8 activity with Fluor-de-Lys substrate.

		Activity (pmol min^-1^ μg^-1^)			
Buffer	Enzyme preparation	No N-acetylthiourea	10 μmol L^-1^ TM-2-51	10 μmol L^-1^ TM-2-88	10 μmol L^-1^ TM-2-104
Assay buffer 1	Co^2+^ resin, cleaved His_6_	3.7 ± 0.4	4.5 ± 0.4	4.4 ± 0.9	4.4 ± 0.3
Assay buffer 2	Co^2+^ resin, cleaved His_6_	18.2 ± 3.4	21.9 ± 4.3	21.0 ± 2.4	17.8 ± 2.7
Assay buffer 2	Ni^2+^ resin, His_6_ tagged	5.3 ± 1.4	4.9 ± 1.0	4.6 ± 1.0	4.5 ± 1.3
Assay buffer 1	Commercial, His_6_ tagged	0.35 ± 0.04	0.68 ± 0.14	0.81 ± 0.26	0.54 ± 0.11

**Fig 2:**
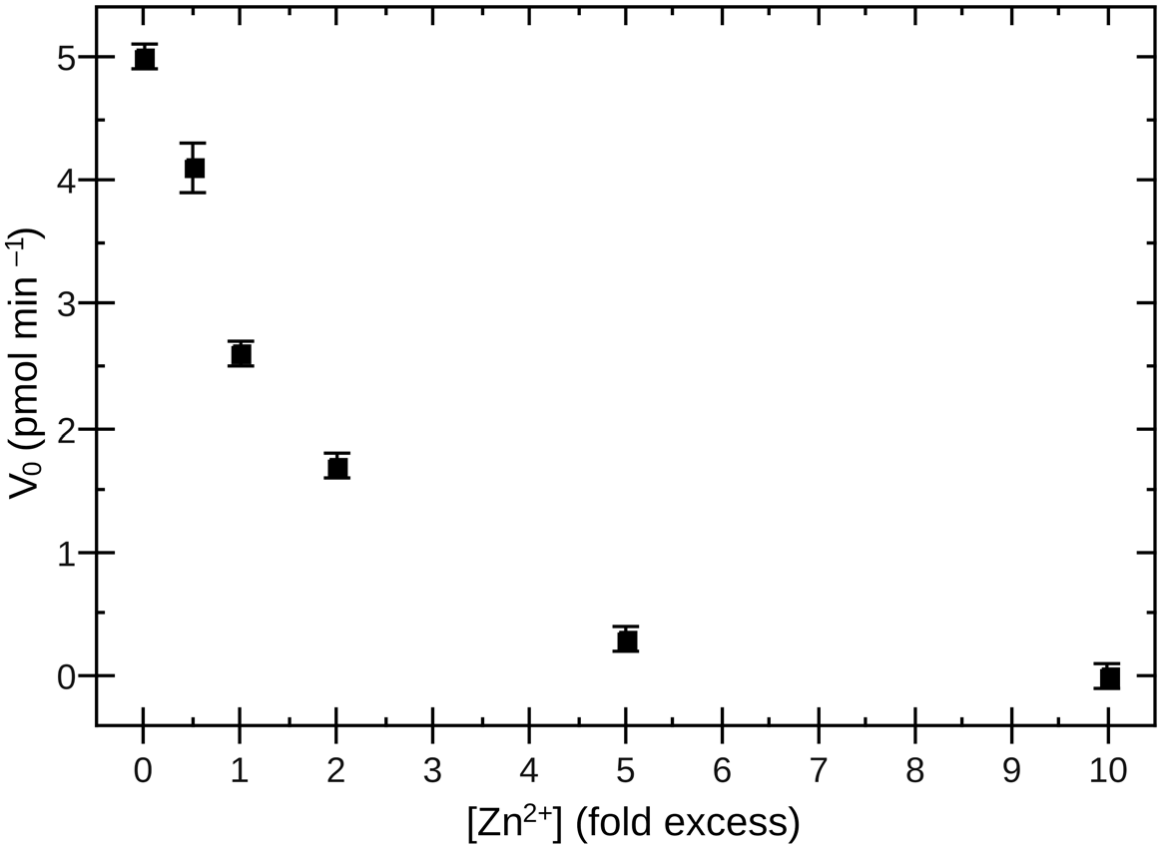
Effect of Zn^2+^ on activity of KDAC8. KDAC8 was incubated with Fluor-de-Lys substrate in assay buffer 3 with 0-10X excess Zn^2+^, as detailed in the Experimental section. The rapid decline in activity indicates that even a small amount of excess zinc is binding to the low affinity inhibitory site, indicating active site saturation. Error bars represent the standard deviation of technical triplicates.

It has previously been reported that KDAC8 is inhibited by excess concentrations of zinc (i.e., addition of zinc beyond the concentration necessary to saturate the active site).[2] We considered that the N-acetylthioureas could potentially activate KDAC8 by reversing this inhibition, and therefore tested the effect of the molecules in the presence of excess Zn^2+^. There was no effect of the N-acetylthioureas in the presence of excess Zn^2+^ under these conditions (Table 3). However, we were also surprised to observe no significant inhibition by zinc independent of the N-acetylthioureas, and so performed a titration of Zn^2+^ in assay buffer 3, which more closely resembled the conditions used when zinc inhibition was reported.[2] KDAC8 does show the expected zinc inhibition in this buffer (Fig 2). It is likely that a composition change in the two buffers, such as overall salt concentration or the presence of BSA, causes this difference in zinc inhibition. In contrast, titrating in excess cobalt had no effect on the enzyme activity in any assay buffer, as expected.[2] Together, the results of the zinc and cobalt titrations indicate that our preparation of KDAC8 results in a protein with an active site saturated with metal. Regardless, Table 3 clearly shows that the N-acetylthioureas still have no effect in the presence of excess zinc under the conditions previously reported.[17,18]

**Table 3:** Effect of N-acetylthioureas on KDAC8 Activity with Fluor-de-Lys substrate in assay buffer 2 in the presence of excess zinc.

	Activity (pmol min^-1^ μg^-1^)			
Concentration of zinc (fold excess)	No N-acetylthiourea	10 μmol L^-1^ TM-2-51	10 μmol L^-1^ TM-2-88	10 μmol L^-1^ TM-2-104
0 nmol L^-1^ (0X)	18.2 ± 3.4	21.9 ± 4.3	21.0 ± 2.4	17.8 ± 2.7
200 nmol L^-1^ (1X)	18.5 ± 1.1	19.7 ± 2.1	18.2 ± 0.9	18.1 ± 0.3
400 nmol L^-1^ (2X)	17.6 ± 1.9	21.7 ± 4.0	19.4 ± 1.7	20.1 ± 4.4

**Fig 3:**
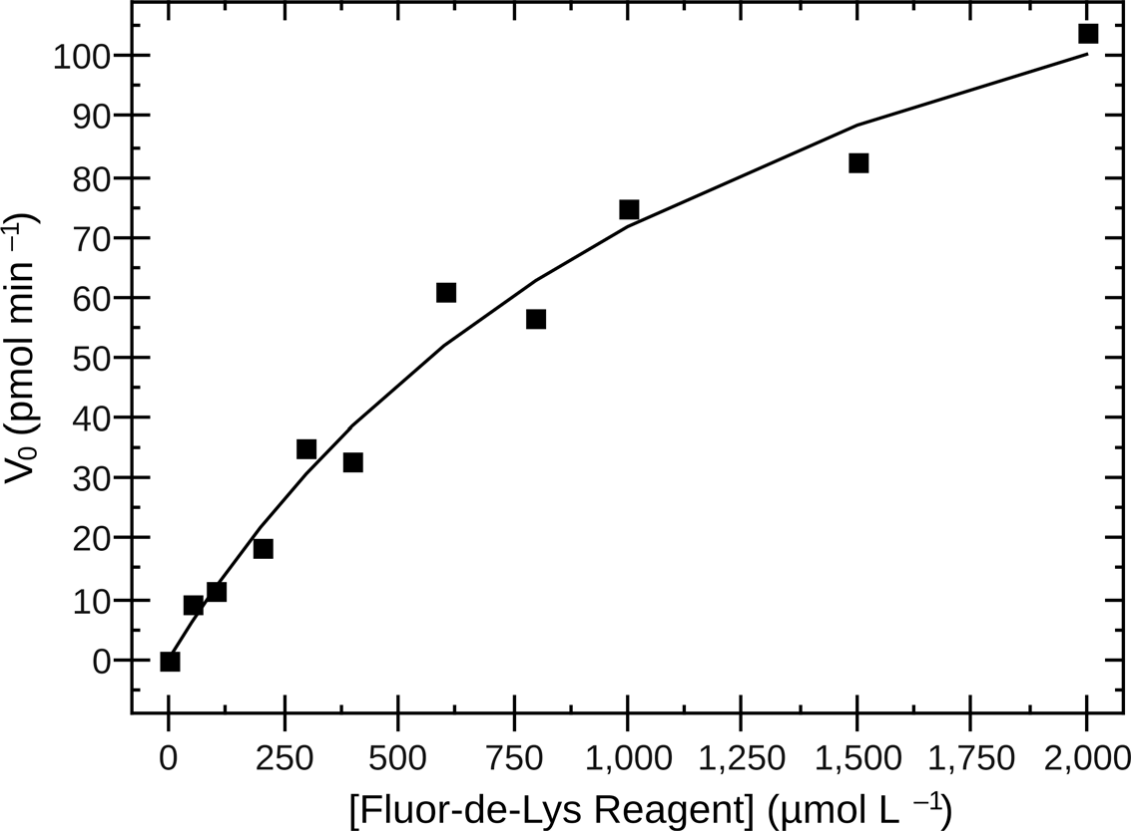
Steady-state kinetics of KDAC8 with Fluor-de-Lys substrate. KDAC8 was incubated with several concentrations of Fluor-de-Lys substrate in assay buffer 2. Aliquots were removed and stopped at various timepoints to determine the initial deacetylation rate for each reaction. Concentration was plotted against initial rate and non-linearly fit to the Michaelis-Menten equation. Steady-state kinetics parameters (Table 4) were calculated from these data.

We also determined the steady-state parameters for KDAC8 with the Fluor-de-Lys substrate (Table 4, Fig 3). Our results indicate about a two-fold lower substrate affinity than that previously reported.[18] Our observed k_cat_, without any N-acetylthiourea, is three-fold higher than that previously reported for the activated KDAC8 and approximately 30-fold higher than previously reported for the enzyme in the absence of the N-acetylthioureas (Table 4). In addition, the catalytic efficiency of our preparation of the enzyme is equal to or greater than that previously reported for the enzyme with N-acetylthiourea TM-2-51 (Table 4).

**Table 4:** Steady-state kinetics parameters for Fluor-de-Lys substrate with KDAC8 in assay buffer 2.

N-acetylthiourea	k_cat_ (s^-1^)	K_M_ (μmol L^-1^)	k_cat_/K_M_ (L mol^-1^ s^-1^)
None ^a^	0.208 ± 0.017	1270 ± 190	164 ± 17
None ^b^	0.007	744	9.4
None ^c^	0.007 ± 0.001	650 ± 20	10.8 ± 1.6
Saturating TM-2-51 ^b^	0.036	230	150
10 μmol L^-1^ TM-2-51 ^c^	0.072 ± 0.014	580 ± 26	124 ± 25

^a^ This work.

^b^ From reference [17].

^c^ From reference [18].

Independent use of 100 μmol L^-1^ of the N-acetylthiourea TM-2-51 was recently reported with a range of KDAC8 variants with the Fluor-de-Lys HDAC8 substrate.[21,22] In all cases, 1.5- to 3-fold activation was reported, similar to what we observed with the commercial KDAC8 preparation. We attempted to replicate these results using our enzyme preparation and identical reaction conditions. Again no activation was observed with our enzyme preparation (Table 5). Our basal activity with the Fluor-de-Lys HDAC8 substrate without TM-2-51 was also substantially higher than the activity previously reported with the N-acetylthiourea, similar to the difference observed between our preparation and the commercial preparation.

**Table 5:** Effect of TM-2-51 on KDAC8 activity with Fluor-de-Lys HDAC8 substrate in assay buffer 4.

	Activity (pmol min^-1^ μg^-1^)	
Source	No N-acetylthiourea	100 μmol L^-1^ TM-2-51
This work	450 ± 50	430 ± 130
Reference [21] ^a^	28 ± 1	66 ± 2
Reference [22] ^a^	27 ± 1	56 ± 2
Reference [2] ^b^	820 ± 140	Not reported

^a^ Converted from the units originally reported using the molecular mass of our KDAC8.

^b^ Calculated from reported k_cat_ and K_M_ values. Reaction temperature was 37°C, which has been previously reported to approximately double the reaction rate compared to 25°C.[19]

## Discussion

The mechanism of activation by small molecules of lysine deacetylases is not fully understood, and has been the focus of considerable controversy (reviewed in [23]). The most potent natural product activator for any lysine deacetylase identified to date, resveratrol, has effects that are strongly substrate-dependent and most effective with unnatural dye-labeled substrates such as Fluor-de-Lys.[23–25] We were interested in whether the same effect was observed with activators of KDAC8, as the interaction between substrate and activator would lead to greater insight into the biological activity of the enzyme.

Our initial screening using a label-free assay system with peptides known to be substrates for KDAC8 revealed no increase in activity by N-acetylthioureas (Table 1). However, our reaction conditions were also different than those used to identify N-acetylthioureas as activating molecules, and so we sought to determine whether the lack of activation was due to substrate-specific effects or buffer-specific effects. Unexpectedly, the results indicated that three of the N-acetylthioureas previously described as the most potent KDAC8 activators failed to activate our KDAC8 preparation even under the original reaction conditions and with the same substrate (Table 2). To further eliminate possible sources of variability, we prepared multiple batches of enzyme, including one under purification conditions that more closely reproduced the original conditions. The motivation was that because the enzyme is metal-dependent, it is possible that the metal content of the enzyme was influenced by the particular type of metal affinity chromatography utilized and/or the presence of the His_6_ tag. Modification of the purification conditions reduced the overall enzyme activity approximately four-fold (Table 2), consistent with some introduction of Ni^2+^ into the enzyme,[2] but did not generate conditions where activation was observed. It is also possible that some impurities present from the nickel-purified protein contribute to the apparent reduction in activity compared to the cobalt-purified/tag-cleaved enzyme. Even lower basal activity was observed with a His_6_-tagged commercial preparation of KDAC8. However, with the commercial preparation, up to 2-fold activation was observed (Table 2), although this still far below the 5- to 20-fold activation previously reported.[17,18] Somewhat less activation was observed with TM-2-104, consistent with the prior report that this was the least potent of these three N-acetylthioureas.[17] This observation of preparation-dependent activation indicates that activation of N-acetylthioureas is dependent on some variation in enzyme preparation.

An alternate mechanism by which metals might play a role in apparent activation is that Zn^2+^ is known to inhibit KDAC8 when present in excess by binding to a second site in the protein.[2] To test the hypothesis that N-acetylthioureas might be “activating” KDAC8 by reversing excess metal inhibition, we also determined the effect of the N-acetylthioureas in the presence of excess zinc. However, the buffer used to originally demonstrate activation, assay buffer 2, eliminates the inhibition by Zn^2+^ (compare Table 3 and Fig 2), perhaps due to non-specific metal binding by BSA attributable to the relatively large excess of BSA compared to enzyme and zinc. Given the lack of inhibition by Zn^2+^ in assay buffer 2, it is not surprising that we did not observe any reversal of metal inhibition by the N-acetylthioureas (Table 3).

To rule out problems with our enzyme preparation, we sought to compare the activity of our enzyme directly to those values previously reported with the N-acetylthioureas.[17,18] Unfortunately, most of the activity measurements for these experiments were reported in terms of fluorescence units, which is instrument-dependent and therefore not comparable between laboratories, or as normalized fold-activation. However, steady-state parameters of KDAC8 with Fluor-de-Lys in the absence and presence of one of the N-acetylthioureas, TM-2-51, have been reported.[18] We obtained steady-state parameters for our preparation of KDAC8 with the Fluor-de-Lys substrate under identical conditions in the absence of any N-acetylthiourea. Interestingly, Singh and coworkers report a large increase in k_cat_ in the presence of TM 2–51. Our calculated k_cat_ in the absence of any N-acetylthiourea is significantly greater than the “activated” k_cat_ that they report, and our catalytic efficiency is at least as high as the reported “activated” efficiency (Table 4). Therefore, our preparation of enzyme may be insensitive to the N-acetylthioureas because it is already operating at the maximum rate, so no additional activation is possible. It is probable that our much higher k_cat_ is due to differences in the amount and/or type of metal contained in the enzyme (Ni^2+^, Zn^2+^, or Co^2+^), with the Ni^2+^ enzyme expected to be slower than the others.[2] Although our observed K_M_ is approximately twice that previously reported,[18] our kinetic parameters are well within the range of values previously reported for similar coumarin-labeled substrates.[2,26,27] Moreover, previous data indicate that the K_M_ is sensitive to the metal present in the active site,[2] so the difference in K_M_ values may also indicate a difference in enzyme metal content.

Further evidence that activation may depend on the baseline activity of the enzyme preparation comes from a comparison to recent reports by an independent research group of activation by TM-2-51. In these reports, KDAC8 prepared similarly to our method was shown to be activated up to approximately 2-fold by up to 100 μmol L^-1^ TM-2-51. A direct comparison of the activity of our enzyme preparation with these reported conditions again confirms that we do not see activation with our enzyme preparation, even using the Fluor-de-Lys HDAC8 substrate and the higher concentration of TM-2-51 (Table 5). However, as in the other cases, our basal activity is also higher than the “activated” level reported. Therefore, analysis of independent preparations of enzyme, reaction conditions, and substrates reveals that lower basal enzyme activity correlates with greater activation by N-acetylthioureas: the enzyme with the lowest basal activity, reported by Singh et. al., was activated up to 10-fold more than the preparations with intermediate activity, and no activation is observed with our preparations with the highest basal activity. Critically, in no case does an “activated” preparation exceed the highest basal activity (i.e., in the absence of any N-acetylthiourea) that we observed, and our high activity preparation results in activity data that is comparable to other reports in the literature (Table 5 and discussed for other substrates in ref [19]).

Therefore, we conclude that the reported activation of KDAC8 by N-acetylthioureas is likely to be due to a specific feature of the preparation of the enzyme rather than a general mechanism, and any biological effects of N-acetylthioureas are unlikely to be due to interactions with KDAC8. In the initial report, it was noted that the N-acetylthioureas had no effect or slight inhibition with all tested KDACs except KDAC8.[17] Our preparation of KDAC8 therefore exhibits behavior consistent with all other KDACs, suggesting that the apparent activation of KDAC8 by the N-acetylthioureas is a function of the specific method used to prepare that enzyme instead of a true activation. It is possible that this preparation effect may have a correlation with the metal content of the enzyme, and that the N-acetylthioureas only serve as activators when the enzyme is partially metal-depleted (i.e., consistent with the lower baseline k_cat_ reported by Singh and coworkers) and that even the fully “active” enzyme contains Ni^2+^ and exhibits sub-optimal activity.[18] However, in the absence of reported data evaluating metal content for enzyme preparations that show activation by the N-acetylthioureas, such as direct determination of metal content by ICP-MS or indirect demonstration of metal saturation by zinc titrations, we are unable to test this hypothesis. Alternatively, perhaps N-acetylthioureas help stabilize a particular conformation of KDAC8 that is more active, and the enzyme preparation conditions influence how much of this conformation is present. Although we have not identified a specific mechanism of action, it is clear that the activation effect of N-acetylthioureas is preparation-dependent and should more appropriately be described as partially reversing a deficiency in the basal activity than true activation.

## Supporting Information

S1 Fig Purified KDAC8. (A) 1 μg KDAC8 purified as described in materials and methods was subjected to SDS-PAGE and stained with GelCode Blue. Center lane is the enzyme purified using cobalt resin following tag cleavage with tobacco etch virus protease, with an expected mass of 42.5 kDa. Right lane is the enzyme purified using nickel resin and without cleaving the tag, as described in the experimental section, with an expected mass of 43.4 kDa. Under the staining conditions, all bands of at least 10 ng would appear. The cobalt-purified protein is substantially more pure than the nickel-purified protein. (B) 1 μg of an independent preparation of KDAC8 purified with cobalt resin (center) and 1 μg of commercial KDAC8. The purity of our KDAC8 is consistent across batches, and is at least as high as that of the commercial preparation (expected mass 46.4 kDa). Equivalent intensity in the two lanes on this gel indicates that enzyme concentrations determined by spectroscopic methods are internally consistent.
(PDF)

S1 Table Comparison of expected and measured ^1^H-NMR spectrum for TM-2-51. (PDF)

S2 Table Comparison of expected and measured ^1^H-NMR spectrum for TM-2-88. (PDF)

S3 Table Comparison of expected and measured ^1^H-NMR spectrum for TM-2-104. (PDF)

S4 Table Comparison of expected and measured melting point temperatures of the N-acetylthioureas. (PDF)
