# Supplementary material for: KDAC8 with High Basal Velocity Is Not Activated by N-Acetylthioureas
Source: PLoS One. 2016 Jan 8;11(1):e0146900. doi: 10.1371/journal.pone.0146900 (PMC4706426; doi:10.1371/journal.pone.0146900)
Supplement: S1 Fig — (A) 1 μg KDAC8 purified as described in materials and methods was subjected to SDS-PAGE and stained with GelCode Blue. Center lane is the enzyme purified using cobalt resin following tag cleavage with tobacco etch virus protease, with an expected mass of 42.5 kDa. Right lane is the enzyme purified using nickel resin and without cleaving the tag, as described in the experimental section, with an expected mass of 43.4 kDa. Under the staining conditions, all bands of at least 10 ng would appear. The cobalt-purified protein is substantially more pure than the nickel-purified protein. (B) 1 μg of an independent preparation of KDAC8 purified with cobalt resin (center) and 1 μg of commercial KDAC8. The purity of our KDAC8 is consistent across batches, and is at least as high as that of the commercial preparation (expected mass 46.4 kDa). Equivalent intensity in the two lanes on this gel indicates that enzyme concentrations determined by spectroscopic methods are internally consistent. (PDF) [file pone.0146900.s001.pdf]

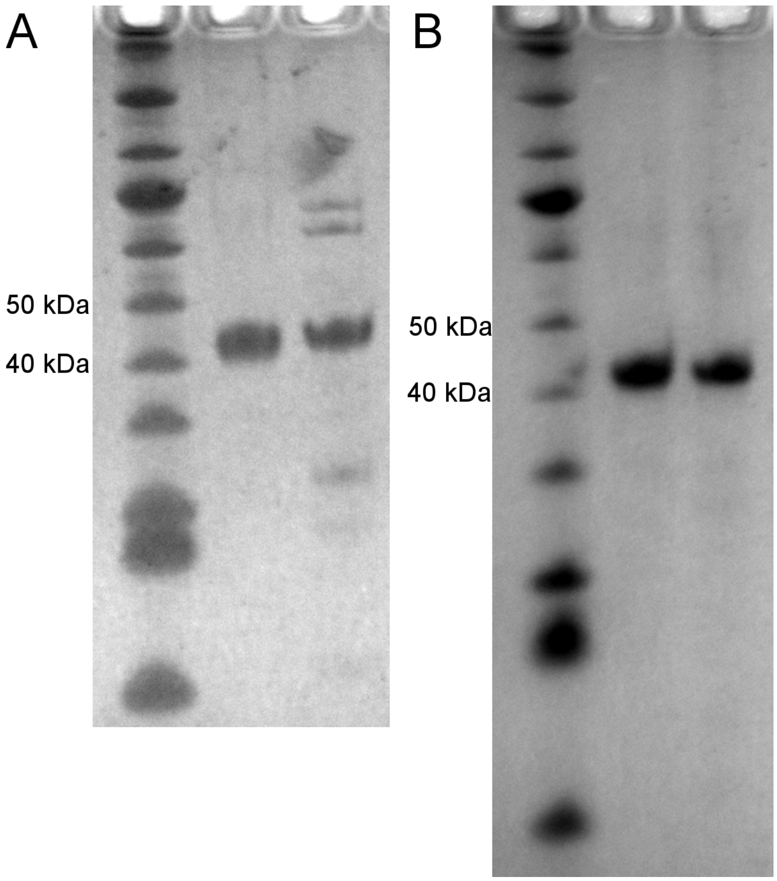

S1 Fig. Purified KDAC8. (A) 1 µg KDAC8 purified as described in materials and methods was subjected to SDS-PAGE and stained with GelCode Blue. Center lane is the enzyme purified using cobalt resin following tag cleavage with tobacco etch virus protease, with an expected mass of 42.5 kDa. Right lane is the enzyme purified using nickel resin and without cleaving the tag, as described in the experimental section, with an expected mass of 43.4 kDa. Under the staining conditions, all bands of at least 10 ng would appear. The cobalt-purified protein is substantially more pure than the nickel-purified protein. (B) 1 µg of an independent preparation of KDAC8 purified with cobalt resin (center) and 1 µg of commercial KDAC8. The purity of our KDAC8 is consistent across batches, and is at least as high as that of the commercial preparation (expected mass 46.4 kDa). Equivalent intensity in the two lanes on this gel indicates that enzyme concentrations determined by spectroscopic methods are internally consistent.
