# Supplementary material for: KDAC8 with High Basal Velocity Is Not Activated by N-Acetylthioureas
Source: PLoS One. 2016 Jan 8;11(1):e0146900. doi: 10.1371/journal.pone.0146900 (PMC4706426; doi:10.1371/journal.pone.0146900)
Supplement: S1 Table — (PDF) [file pone.0146900.s002.pdf]

S1 Table. Comparison of expected and measured ^{1}H-NMR spectrum for TM-2-51.

Proton(s)	Expected δ (ppm) [a] Acetone-D_6, 400 MHz	Measured δ (ppm) Acetone-D_6, 300 MHz
N	- [b]	- [b]
N’	10.3 (bs, 1H)	- [b]
C3, C7	8.06 - 8.04 (m, 2H)	8.11 - 8.08 (m, 2H)
C2’, C6’	7.78 - 7.76 (m, 2H)	7.84 - 7.81 (m, 2H)
C5	7.69 - 7.64 (m, 1H)	7.72 - 7.69 (m, 1H)
C4, C6	7.57 - 7.53 (m, 2H)	7.63 - 7.60 (m, 2H)
C3’, C5’	7.43 - 7.38 (m, 2H)	7.48 - 7.45 (m, 2H)
C4’	7.28 - 7.24 (m, 1H)	7.32 - 7.27 (m, 1H)

[a] From reference [17].
[b] Not observed.
