# Supplementary material for: KDAC8 with High Basal Velocity Is Not Activated by N-Acetylthioureas
Source: PLoS One. 2016 Jan 8;11(1):e0146900. doi: 10.1371/journal.pone.0146900 (PMC4706426; doi:10.1371/journal.pone.0146900)
Supplement: S2 Table — (PDF) [file pone.0146900.s003.pdf]

S2 Table. Comparison of expected and measured ^{1}H-NMR spectrum for TM-2-88.

Proton(s)	Expected δ (ppm) [a] Acetone-D_6, 300 MHz	Measured δ (ppm) Acetone-D_6, 300 MHz
N	12.86 (bs, 1H)	12.87 (bs, 1H)
N’	10.27 (bs, 1H)	10.27 (bs, 1H)
C3, C7	8.08 (d, J = 7.8 Hz, 2H)	8.10 (d, 2H)
C4, C5, C6	7.73 - 7.56 (m, 3H)	7.74 - 7.59 (m, 3H)
C2’, C6’	7.11 (d, J = 2.4 Hz, 2H)	7.13 (s, 2H)
C4’	6.42 - 6.40 (m, 1H)	6.43 - 6.41 (m, 1H)
Methoxy	3.81 (s, 6H)	3.82 (s, 6H)

[a] From reference [17].
