# Supplementary material for: KDAC8 with High Basal Velocity Is Not Activated by N-Acetylthioureas
Source: PLoS One. 2016 Jan 8;11(1):e0146900. doi: 10.1371/journal.pone.0146900 (PMC4706426; doi:10.1371/journal.pone.0146900)
Supplement: S3 Table — (PDF) [file pone.0146900.s004.pdf]

S3 Table. Comparison of expected and measured ^{1}H-NMR spectrum for TM-2-104.

Proton(s)	Expected δ (ppm) [a]<br>$CDCl_3$, 400 MHz	Measured δ (ppm)<br>$CDCl_3$, 300 MHz
N	10.90 (d, J = 6.4 Hz, 1H)	11.02 (d, 1H)
N’	8.75 (bs, 1H)	9.02 (bs, 1H)
C3, C7	7.63 - 7.61 (m, 2H)	7.70 - 7.67 (m, 2H)
C5	7.43 - 7.39 (m, 1H)	7.48 - 7.43 (m, 1H)
C5’, C7’	7.33 - 7.28 (m, 2H)	7.36 - 7.31 (m, 2H)
C4, C6, C4’, C8’	7.21 - 7.15 (m, 4H)	7.25 - 7.23 (m, 4H)
C6’	7.11 - 7.07 (m, 1H)	7.17 - 7.11 (m, 1H)
C2’	5.45 - 5.38 (m, 1H)	5.54 - 5.44 (m, 1H)
C1’	1.46 (d, J = 6.8 Hz, 3H)	1.53 (d, 3H)

[a] From reference [17].
