# Supplementary material for: KDAC8 with High Basal Velocity Is Not Activated by N-Acetylthioureas
Source: PLoS One. 2016 Jan 8;11(1):e0146900. doi: 10.1371/journal.pone.0146900 (PMC4706426; doi:10.1371/journal.pone.0146900)
Supplement: S4 Table — (PDF) [file pone.0146900.s005.pdf]

S4 Table. Comparison of expected and measured melting point temperatures of the N-acetylthioureas.

	Melting point (°C)	
N-acetylthiourea	Expected [a]	Measured
TM-2-51	148 - 150	144 - 146
TM-2-88	154 - 156	152 - 155
TM-2-104	- [b]	- [b]

[a] From reference [17].
[b] Not measured, as the melting temperature is below room temperature.
